# Nanometric Moiré Stripes on the Surface of Bi_2_Se_3_ Topological Insulator

**DOI:** 10.1021/acsnano.2c02515

**Published:** 2022-09-13

**Authors:** Matteo Salvato, Maurizio De Crescenzi, Mattia Scagliotti, Paola Castrucci, Simona Boninelli, Giuseppe Mario Caruso, Yi Liu, Anders Mikkelsen, Rainer Timm, Suhas Nahas, Annica Black-Schaffer, Gunta Kunakova, Jana Andzane, Donats Erts, Thilo Bauch, Floriana Lombardi

**Affiliations:** † Dipartimento di Fisica and INFN, Università di Roma “Tor Vergata”, 00133 Roma, Italy; ‡ CNR-IMM, Strada VIII 5, 95121 Catania, Italy; § Division of Synchrotron Radiation Research, Department of Physics and NanoLund, Lund University, 221 00 Lund, Sweden; ∥ Department of Physics and Astronomy, Uppsala University, Box 516, 75120 Uppsala, Sweden; ⊥ Institute of Chemical Physics, University of Latvia, LV-1586 Riga, Latvia; # Quantum Device Physics Laboratory, Department of Microtechnology and Nanoscience, Chalmers University of Technology, 41296 Goteborg, Sweden

**Keywords:** topological insulators, Bi_2_Se_3_, van der Waals epitaxy, moiré stripes, local density of states

## Abstract

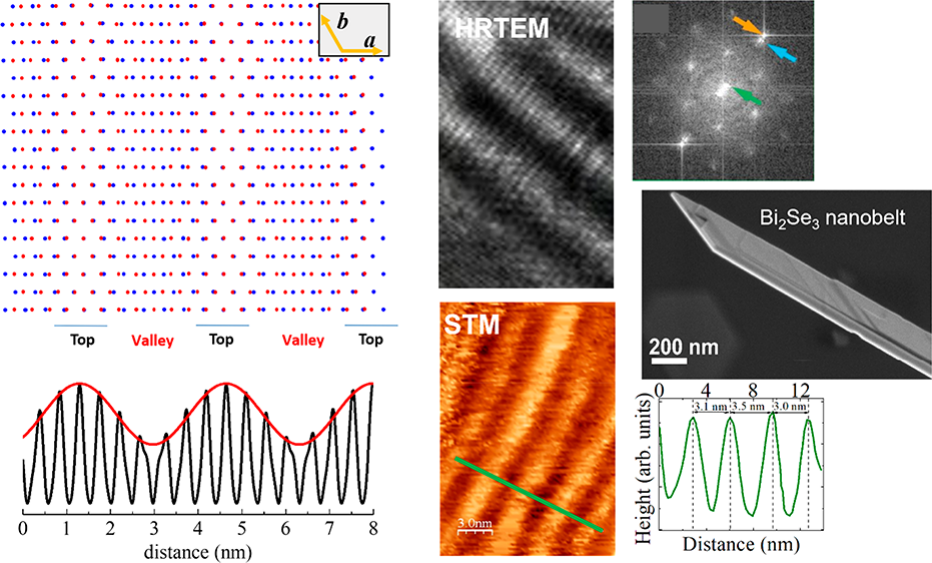

Mismatch between adjacent atomic layers in low-dimensional materials, generating moiré patterns, has recently emerged as a suitable method to tune electronic properties by inducing strong electron correlations and generating novel phenomena. Beyond graphene, van der Waals structures such as three-dimensional (3D) topological insulators (TIs) appear as ideal candidates for the study of these phenomena due to the weak coupling between layers. Here we discover and investigate the origin of 1D moiré stripes on the surface of Bi_2_Se_3_ TI thin films and nanobelts. Scanning tunneling microscopy and high-resolution transmission electron microscopy reveal a unidirectional strained top layer, in the range 14–25%, with respect to the relaxed bulk structure, which cannot be ascribed to the mismatch with the substrate lattice but rather to strain induced by a specific growth mechanism. The 1D stripes are characterized by a spatial modulation of the local density of states, which is strongly enhanced compared to the bulk system. Density functional theory calculations confirm the experimental findings, showing that the TI surface Dirac cone is preserved in the 1D moiré stripes, as expected from the topology, though with a heavily renormalized Fermi velocity that also changes between the top and valley of the stripes. The strongly enhanced density of surface states in the TI 1D moiré superstructure can be instrumental in promoting strong correlations in the topological surface states, which can be responsible for surface magnetism and topological superconductivity.

In recent years, the ability to control a small misalignment angle between stacks of 2D materials has been extensively employed to generate moiré patterns, strongly affecting the electronic properties of the underlying material.^1−3^ Bilayer graphene is certainly one of the most striking examples, where a “magic” twist angle between two layers creates weakly dispersive “flat” bands, promoting strong electron–electron correlations. This facilitates the emergence of strongly correlated phases, such as superconductivity,^4^ magnetism,^5^ and insulating states.^6^ In particular, magic angle bilayer graphene presents a phase diagram with many analogies to the high-temperature cuprate superconductors.^7^ However, the fine-tuning required to obtain the magic angle poses challenges to the fabrication and scalability of the devices. An alternative route to explore moiré physics, is to introduce strain or buckling in the 2D system. Also in this case, graphene has pioneered the field, and flat bands have been observed when a “buckling” transition was induced in the bare material.^2^

Motivated by the success of twisted van der Waals heterostructures,^1^ recent theoretical approaches have also studied the possibility to promote strongly correlated electronic phases into the surface Dirac electrons of a 3D topological insulator (TI).^8,9^ Here the fundamental difference between the Dirac cone in 3D TI and magic angle graphene, dictated by topology, prevents the formation of an induced moiré miniband in the gap, since it would violate the requirement that extended surface states exist at all energies within the bulk band gap.^9^ Thus, at least theoretically, the Dirac cones on the surface of a 3D TI exhibit fundamentally different behavior in a moiré potential compared to the Dirac cones in magic angle/buckled graphene. Engineering of interacting instabilities on the surface of 3D TIs would therefore give rise to properties such as surface magnetism^10^ and topological superconductivity^11^ that can be profoundly different from other 2D moiré superlattices. Moreover, vortices in the superconducting state would also host the long-sought-after Majorana fermions.^12^ However, the experimental realization of moiré superstructures in 3D TIs cannot follow the traditional path of other 2D materials, because of current experimental difficulties in stacking single layers.

Here we show that the TI Bi_2_Se_3_, in both the form of nanobelts and that of thin films, naturally develops moiré patterns on its surface. We attribute this to a substantial unidirectional tensile strain imposed on the last layer by the specifics of the growth process, and it is notably not induced by the substrate lattice mismatch. As a result, the topmost unit cell is dramatically stretched and buckled, forming peculiar 1D moiré stripes. We reveal the buckled shape of the stripes by scanning tunneling microscopy (STM) and by high-resolution transmission electron microscopy (HRTEM). Scanning tunneling spectroscopy (STS) also shows that the local density of states (LDOS) dramatically changes across the tops and valleys of the buckled moiré stripes. By performing density functional theory (DFT) calculations, we find a dramatic increase in the LDOS within the bulk electronic band gap, in agreement with the experimental findings. In addition, as expected by topological protection, the calculations show the preservation of the Dirac cones across the stripes (although in a reduced energy window close to the Dirac point), but notably with substantial and different renormalizations of the Fermi velocities at the tops and valleys of the moiré stripes.

## Results and Discussion

Bi_2_Se_3_ thin films with thicknesses in the range 1–10 nm, roughly corresponding to 1–10 quintuple layers (QLs), the unit stacking block of TIs along the *c* axis,^13^ were deposited by solid–vapor deposition on Si(001), Pt(111), and amorphous glass substrates (see ref (14) and Methods for specifics on the fabrication and employed substrates). The film thickness was measured by X-ray reflectivity with an accuracy of 0.1 nm (see Figure S1a in the Supporting Information). Figure S1 also shows typical X-ray diffraction spectra of samples deposited on Pt–Si (Figure S1b) and on glass substrates (Figure S1c). The peaks are indexed following the *R*3̅*m* lattice structure. Only the (00*l*) reflections of the Bi_2_Se_3_ phase are present, confirming the *c* axis orientation growth and giving a first indication of the absence of other possible phases in our films. Moreover, the presence of Laue reflections reported in Figure S1e,f confirms the ordered structure of the films along the growth direction, as formed by an integer number of unit cells. This is a strong indication that the Bi_2_Se_3_ lattice structure and orientation are not affected by the substrate lattice, as expected for van der Waals epitaxy, and that the growth proceeds following stacking of QL blocks along the direction perpendicular to the substrate surface.

**Figure 1:**
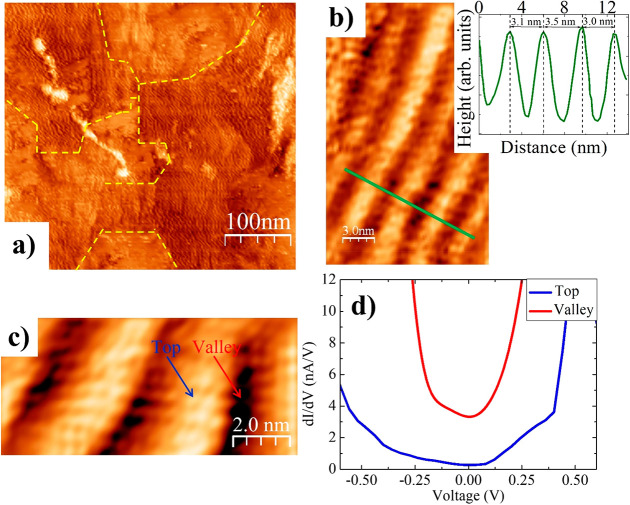
STM and STS on Bi_2_Se_3_ thin films. (a) 0.4 μm × 0.4 μm STM image acquired on a 10 nm thick Bi_2_Se_3_ film deposited on a Si substrate (STM parameters *I* = 0.4 nA, *V* = 0.58 V). Yellow dashed lines are guides for the eye to delimit some striped regions. (b) Enlargement of a 10 nm × 15 nm region in (a) showing stripes (*I* = 0.4 nA, *V* = 0.30 V). The line profile in (b) is reported in the inset, where the distances 3.1, 3.5, and 3.0 nm between adjacent stripes are measured. (c) Enlargement of (b) showing two stripes with atomic resolution (*I* = 0.4 nA, *V* = 0.25 V). The arrows indicate the top and valley regions where the *I–V* characteristics are acquired. (d) d*I*/d*V* vs *V* obtained by averaging the *I–V* measurements reported in Figure S2 acquired in different top and valley regions of the film surface. *V* = 0 corresponds to the Fermi level. *V* bias step: 40 mV.

We further performed STM and STS analyses on Bi_2_Se_3_ films deposited on Si and on Pt substrates under ultrahigh vacuum at room temperature without any treatment of the surface. Figure 1a shows the STM image of an extended area of a 10 nm thick Bi_2_Se_3_ film deposited on Si(001). The whole image consists of flat terraces with long stripes continuously extending for hundreds of nanometers along the same average direction (vertical in the figure). The higher resolution STM image in Figure 1b shows parallel 1D moiré stripes with an average period of 3.2 nm (see inset), as measured by the line profile along the transverse green line in the figure. The measured average period corresponds to a length of between seven and eight lattice parameters of Bi_2_Se_3_. This is estimated by the STM image reported in Figure 1c, which shows two adjacent moiré stripes with a lattice parameter of 0.40 ± 0.05 nm, in good agreement with that expected for Bi_2_Se_3_.^13^ It is worth pointing out that the distance between two adjacent stripes is not constant, as shown in the inset in Figure 1b and by the great number of measurements performed on different STM patterns and reported as statistics in Figure S2b,c for films deposited on both substrates. These statistics give 3.0 ± 0.6 and 2.6 ± 0.7 nm as the results for the separation between two adjacent moiré stripes in films deposited on Si and Pt substrates, respectively. Figure 1d shows the d*I*/d*V* vs *V* curves, proportional to the LDOS, obtained by the current–voltage (*I–V*) measurements positioning the STM tip in the center of a stripe (top) and between two adjacent stripes (valley), as indicated in Figure 1c. The data are representative of several direct *I–V* measurements (some of them being reported in Figure S3) taken on different areas of the sample surface. The average conductance at the valley of the stripes (red line) is considerably higher than that at the stripes’ top. Similar results are obtained for Bi_2_Se_3_ deposited on a Pt substrate, as reported in Figure S4. In the case of both Si and Pt substrates, moiré patterns were not detected for Bi_2_Se_3_ films thinner than 6 QLs. For films thicker than 6 QLs the d*I*/d*V* curves present a minimum at *V* = 0, indicating that the Fermi level lies at the Dirac point, as expected for stoichiometric TIs.^15^

**Figure 2:**
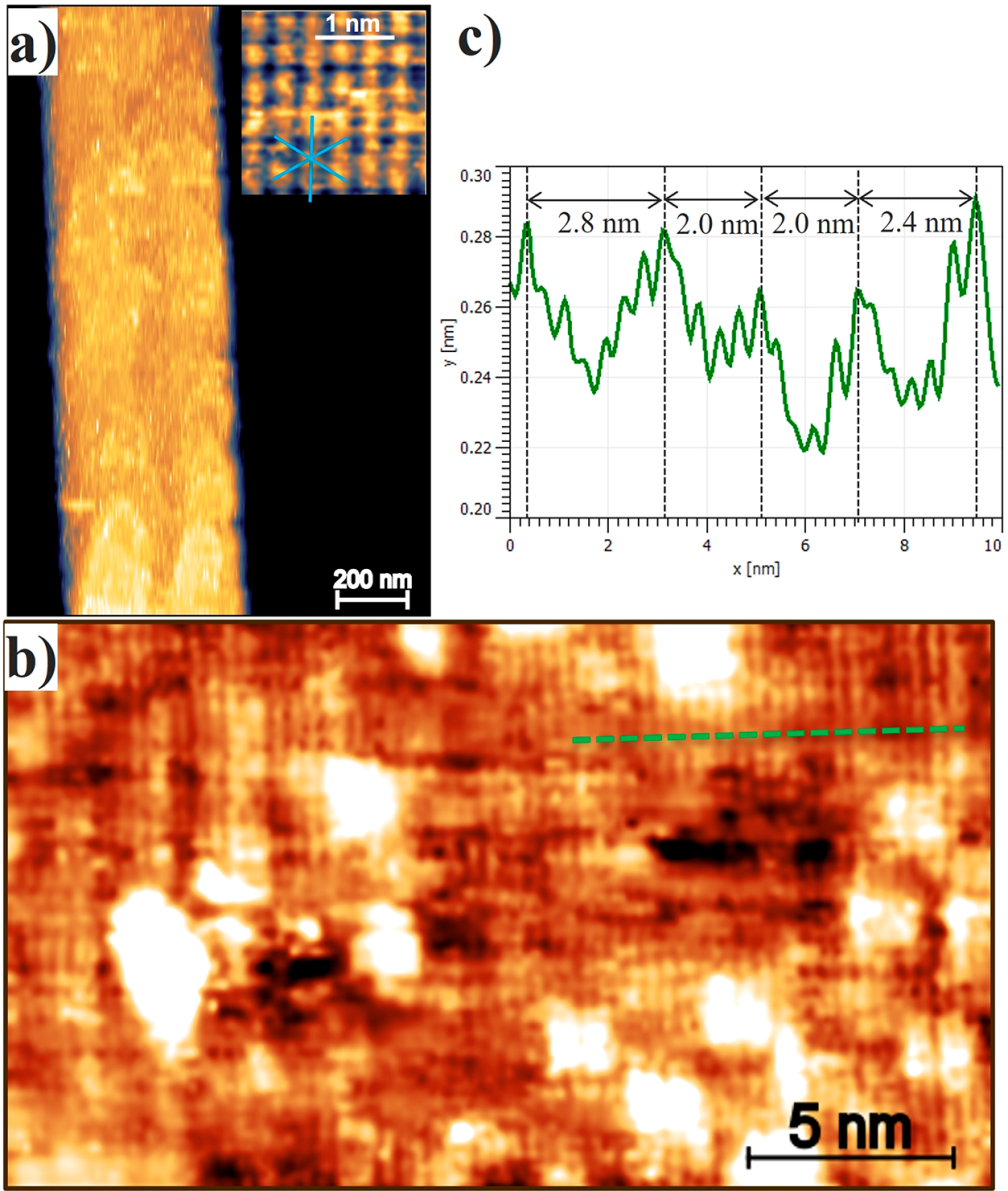
STM images at different resolution of a part of a 40 nm thick Bi_2_Se_3_ nanobelt (a) before and (b) after Ar+ ion sputtering and thermal annealing. The inset in (a) shows a 2 nm × 2 nm area of the same nanobelt after surface oxide removal. The hexagonal symmetry of the surface has been indicated. The bright features in (b) are due to the remaining oxide patches. The line profile (green dashed line) obtained in (b), averaged over a width of 1.8 nm, is reported in (c), showing atomic corrugation and stripes, where the distances between adjacent stripes varies among 5, 6, or 7 in-plane lattice parameters. STM imaging parameters: *V* = 3.0 V, *I* = 40 pA in (a); *V* = 3.0 V, *I* = 30 pA in (b).

In addition to the 1D moiré stripes in thin films, we observe buckled 1D moiré patterns also on the surface of freestanding Bi_2_Se_3_ nanobelts. These are grown by a vapor–solid method,^14,16^ a growth mechanism briefly discussed in the comments to Figure S5a,b, where the crystal-oriented growth is not assisted by the presence of a substrate. Figure 2a shows an STM overview image of a part of a nanobelt after being transferred on a Si substrate. Atomic resolution could be obtained in STM images from sufficiently flat and clean areas of the surface (see Methods), as demonstrated in the inset of Figure 2a, with a lateral distance between the atoms of 0.40 ± 0.05 nm. Figure 2b exhibits the STM image of a restricted region of the nanobelt surface showing parallel stripes along the vertical direction. The distances of the 1D moiré stripes here are 2.0, 2.4, and 2.8 nm, corresponding to five, six, and seven in-plane lattice parameters, respectively. The atomic corrugation and the moiré stripe pattern can clearly be distinguished in height profiles, such as that shown in Figure 2c. The shape of the moiré stripes observed on the nanobelt surface is very similar to that observed in the case of thin films, although the amplitude and the separation are different. This might be due to local inhomogeneity in the surface morphology and strain relaxation in the nanobelts due to different experimental parameters such as growth conditions, surface treatment, and sample resistivity, as well as STM tip shape and applied voltage.

**Figure 3:**
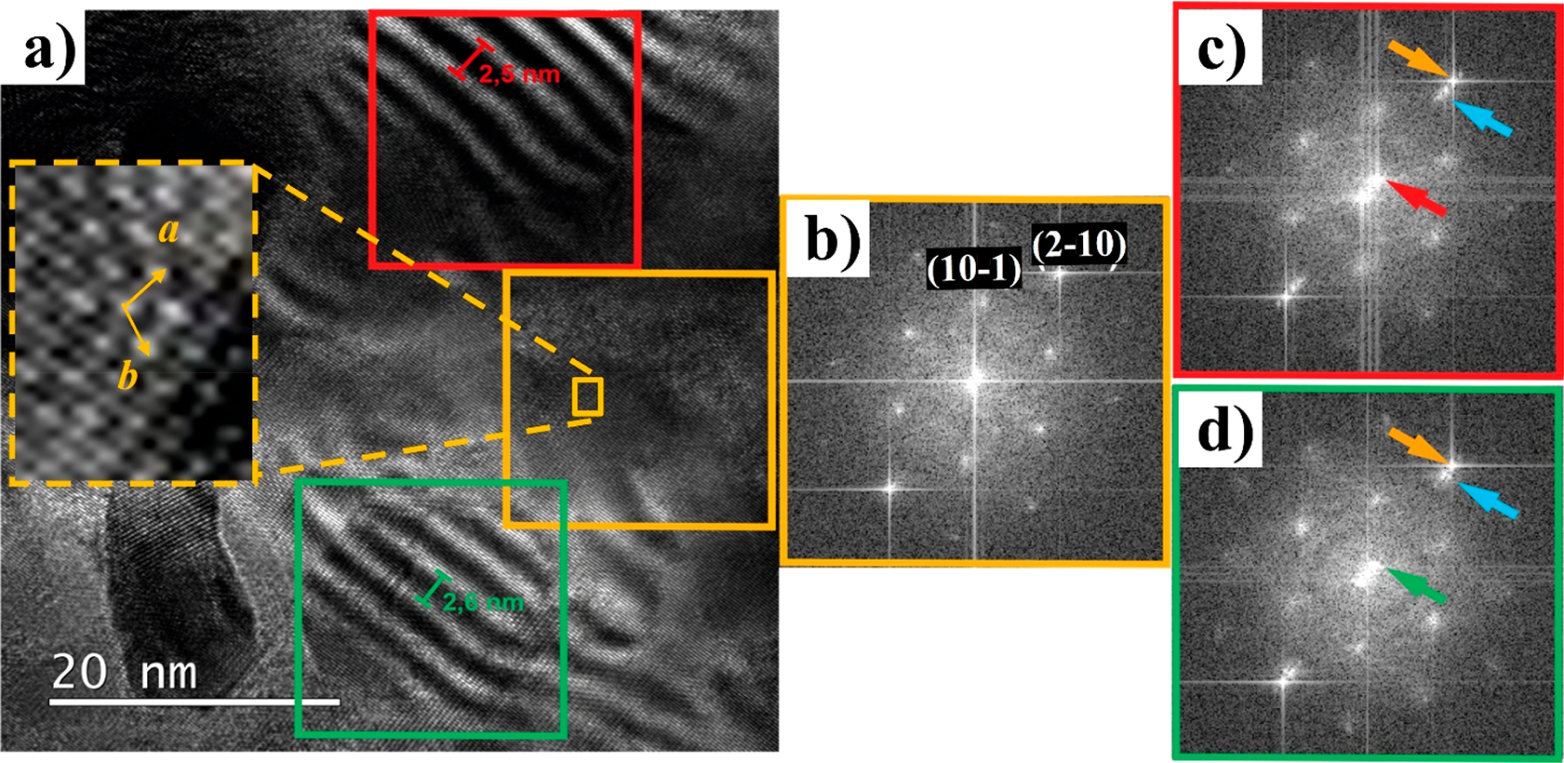
HRTEM and related FFT of a Bi_2_Se_3_ nanobelt. (a) HRTEM micrograph of the surface of a Bi_2_Se_3_ nanobelt. Three squares highlight two regions with stripes and one without any features, as a reference. The dashed box shows an atomic resolution of the region without stripes. (b) FFT of region with no moiré fringes corresponding to the monocrystalline Bi_2_Se_3_. (c, d) FFT shows new features marked by arrows. Red and green arrows indicate the 2.5 and 2.6 nm periodicities of moiré stripes, respectively. The orange arrow indicates the spot corresponding to the (2–10) interplanar distance, while blue arrows indicate a direct lattice strain along the (2–10) direction.

Figure 3a shows an HRTEM micrograph of the Bi_2_Se_3_ nanobelt. We emphasize the presence of periodic moiré stripes (red and green squares), whose interspacing distances are shown in Figure 2c. The area with no stripes (orange square) shows the hexagonal atomic in-plane structure, evidenced in the dashed enlarged square, with a lattice parameter *a* = 0.42 ± 0.01 nm. In Figure 3b–d we compare the fast Fourier transform (FFT) of the HRTEM images highlighted by the colored square boxes in Figure 3a. The presence of the stripes causes some extra spots at the (2–10) reflection, as shown in Figure 3c,d. These spots indicate the overlapping of two plane families with a slightly different interplanar distance: the spot marked by the orange arrow is associated with the 0.21 ± 0.01 nm distance corresponding to the not strained (2–10) planes, while the blue arrow corresponds to a larger distance (0.24 ± 0.01 nm). Since the (2–10) reflections of the reciprocal lattice are along to the ⟨100⟩ ≡ *a* direction of the real lattice, these results show the presence of a 14% tensile strained layer along the *a* direction of the real lattice of the nanobelt. We emphasize here that no extra spots are present along any other direction of the reciprocal lattice, confirming the unidirectional strain of the structure.

Modulated moiré stripes on the surface of TI thin films have been previously reported in the literature and ascribed to several effects, such as chemical doping,^17^ inhomogeneity,^18^ Friedel oscillations,^19^ and substrate mismatch.^20^ In our case, doping can be ruled out due to the absence of any precursor during the growth process and the quasi-perfect stoichiometry obtained by our growth method.^14^ Chemical inhomogeneity can also be ruled out because all possible Bi_*x*_Se_*y*_ compounds have *a*, *b*, and/or *c* axis lengths different from those of Bi_2_Se_3_ or have a different lattice structure,^21^ which would be detectable by a combined X-ray diffraction and HRTEM analysis. In addition, the lack of Se on the top layer can also be excluded because the presence of Se vacancies causes a shift in the position of the Fermi level with respect to the Dirac point^22^ that is not observed in our *dI*/d*V* measurements reported in Figure 1d and Figure S4d. Friedel oscillations, which generate damped stripes moving away from a surface defect, can here be also ruled out, since in our case the modulation appears uniform on areas as large as hundreds of nanometers. Concerning mismatch with the substrate, we observe that, in the case of both Si and Pt substrates, stripes are detected only in our thickest films, while no stripes are observed on Bi_2_Se_3_ films with thicknesses less than 6 QLs, where the effect of the substrate lattice should be more prominent.^23^ Moreover, the contribution of the substrate lattice would be decisive in the case of epitaxial growth. For the Si substrate, epitaxy can only be obtained by a reconstruction of the surface lattice, which is normally performed at high temperature under ultrahigh-vacuum conditions before the film growth, conditions that are not achieved in our deposition system. In addition, the statistics reported in Figure S2b,c give average separations between adjacent stripes of 3.0 ± 0.6 and 2.6 ± 0.7 nm for films deposited on Si and on Pt substrates, respectively. This result gives two important indications: (i) the stripe distance is on average the same, within the experimental uncertainty, despite the different substrate lattices and (ii) such large spreads of ±0.6 and ±0.7 nm in the moiré stripe separation are not very compatible with any epitaxy between the film and the underlayer substrate lattices. But most importantly, the observation of moiré stripes in freestanding Bi_2_Se_3_ nanobelts, where a substrate cannot influence the growth mechanism, definitively rules out the effect of the substrate lattice as the cause of the moiré stripe formation.

**Figure 4:**
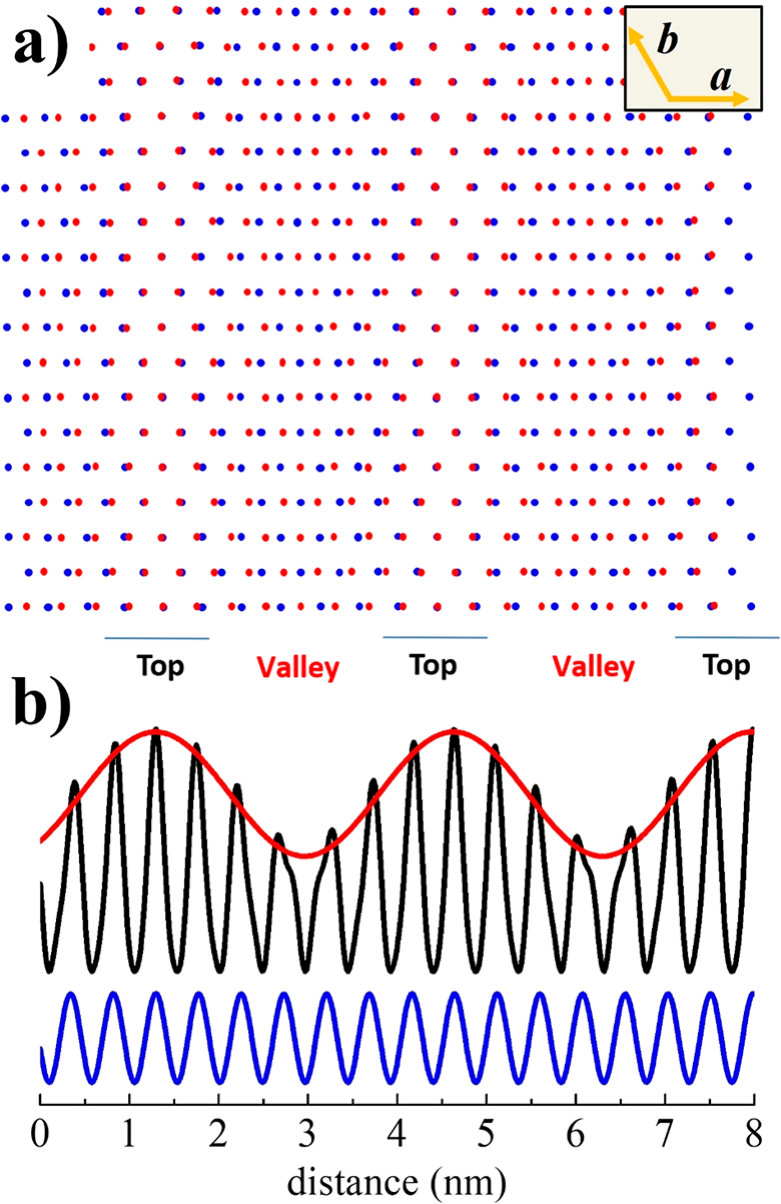
Qualitative simulation and mathematical modeling of 1D moiré stripes. (a) Sketch of two mismatched triangular layers as possible qualitative simulation of the moiré stripe formation. Blue and red dots refer to the atomic positions of the strained outermost and the underlying QLs, respectively. A mismatch of 14% along the transverse direction has been introduced between the two triangular lattices to obtain a periodicity of 3.3 nm (roughly 7–8 lattice spaces). (b) Convolution of the periodic functions representing the lattice periodicity of the two topmost QLs (black line, *z*/*λ* = 1) and the outermost QL and the substrate (blue line, *z*/λ = 6).

Excluding all the aforementioned causes, we developed a simple model simulating the 1D moiré stripes based on an intrinsic mismatch between two adjacent QLs of the Bi_2_Se_3_ crystal structure. This model is shown in Figure 4a, where a topmost layer (blue points), uniaxially strained along the *a* direction, is overlapped to an unstrained underlayer (red points) in a 7 × 8 registry. We emphasize that the uniaxial sketch in Figure 4a is the only possible configuration giving the formation of stripes along *a* with the shape observed in our films and nanobelts. Any different deformation of one of the lattices with respect to the other, such as a twist around the same center point or a uniform strain along both *a* and *b*, would give rise to different moiré patterns (some examples are given in Figure S6). Figure 4b shows the mathematical modeling of the interaction between the two periodic gratings obtained by the expression [1 + cos(*k*_1_*x*)][1 + e^-z/λ^) cos(*k_2_x*)].^24^ This results from the convolution of two periodic functions with wavevectors *k*_1_*= 2*π/0.478 nm^–1^ and *k*_2_ = 2π/0.418 nm^–1^ corresponding to the strained outermost layer (*a* = 0.478 nm) and the underlying layer (*a* = 0.418 nm) of Bi_2_Se_3_, respectively. The factor e^-z/λ^ considers the contribution to the STM signal from a depth *z*,^25^ with λ being the tunnel electron wavelength. Assuming λ to be on the order of the thickness of the topmost QL, the convolution function obtained for *z*/*λ* = 1 (black line in Figure 4b) clearly shows the interference effect of the signals coming from the top surface and the interface with the second QL. It consists of an amplitude-modulated wave (evidenced by the red line in Figure 4b) resembling the top and valley profile of Figures 1b (inset) and 2c with an overall period of 3.3 nm. It is worth noting that the same modeling operating between the outermost QL and the substrate (*z*/*λ* in the range 6–10 for the thickest films) gives a constant amplitude (blue line in Figure 4b) for *z*/*λ* = 6, yet again excluding the possible influence of the substrate lattice on the moiré pattern formation. We would emphasize that the calculated period of 3.3 nm depends on the 7 × 8 registry chosen as a match between the two topmost layers. Different periods can be obtained by changing this matching. For example, the combinations 7 × 8, 6 × 7, 5 × 6, and 4 × 5 in the unit cells of the two topmost layers give moiré stripe periods of 3.3, 2.9, 2.5, and 2.1 nm, respectively, with the corresponding lattice mismatches of 14%, 17%, 20%, and 25%. These periodicities are all within the experimental measurements obtained for the stripes on different samples and reported in Figure S2b,c. This suggests that the spread measured in the stripe periodicity can be ascribed to different combinations in the registry of the two topmost layers in different samples, whose cause will be better discussed below.

**Figure 5:**
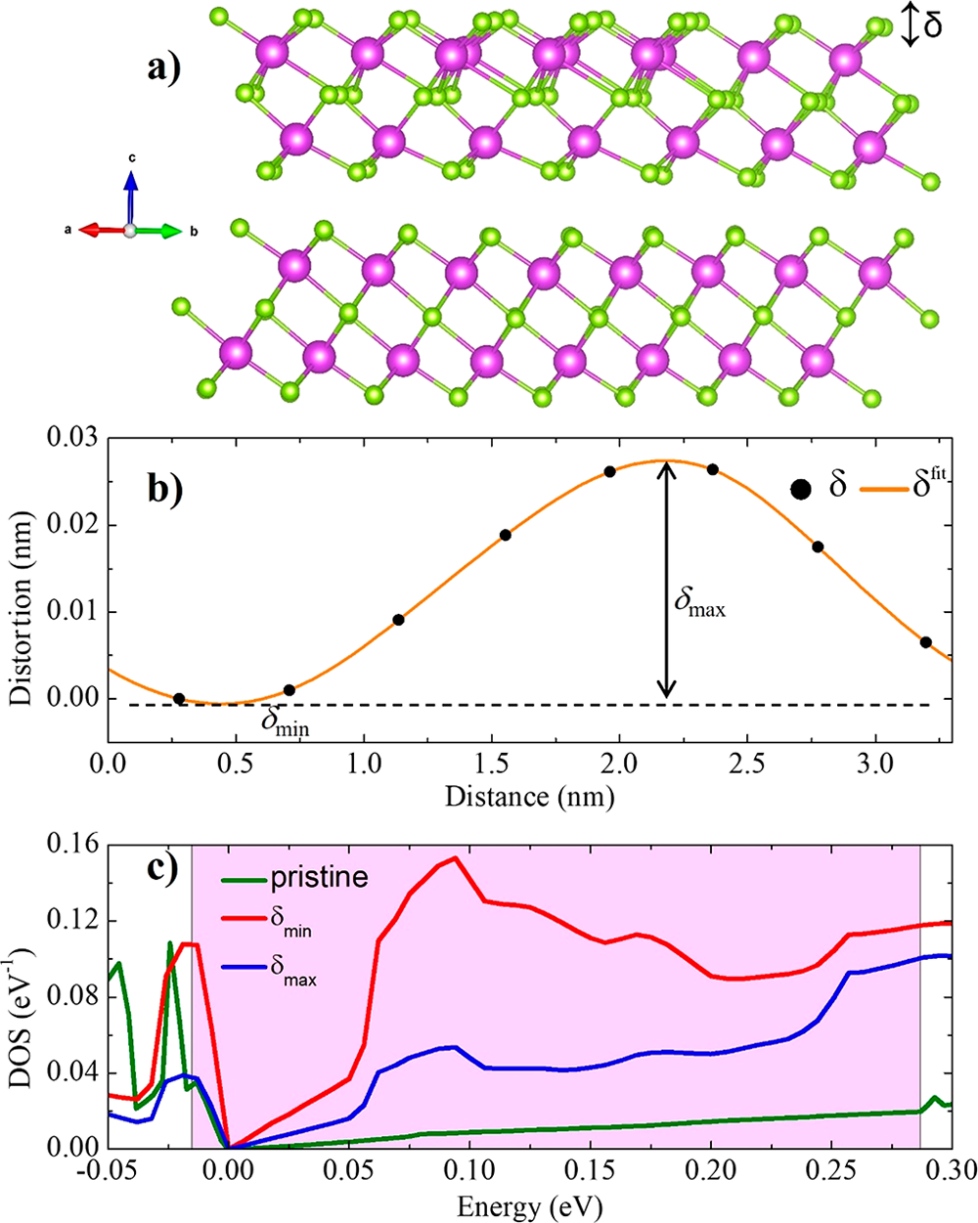
Results from DFT calculations. (a) Side view of the supercell obtained after *ab initio* structural optimization with Bi (Se) atoms in purple (green). The supercell contains in the bulk a period of 8 (1) primitive unit cells in the *a* (*b*) direction, while the surface QL has only 7 (1) primitive unit cells. The out-of-plane *c* direction distortions (δ) of the outermost Se layer in the surface QL is indicated schematically by the vertical arrow (larger than actual distortions). (b) Distortions δ of the atoms in the topmost Se layer of the surface as a function of distance along the *a* direction. The spline fitted curve is denoted by δ^fit^ by a solid orange line. Maximum and minimum distortions of the surface QL are indicated by δ_max_ and δ_min_, respectively. (c) LDOS as a function of energy of the unstrained or pristine surface QL (green) and the corrugated QL taken in the regions δ_max_ (blue) and δ_min_ (red). The bulk gap regions of both the pristine and the striped Bi_2_Se_3_ have been aligned and are indicated by the pink block.

Further insight into the stripe formation comes from an *ab initio* numerical simulation using DFT (see Methods for details). We model the striped system by considering a six-QL-thick slab where we repeat the primitive Bi_2_Se_3_ unit cell along the in-surface *a* direction for eight periods, except for the surface QL, which instead only incorporates seven periods of the primitive Bi_2_Se_3_ unit cell. To find a stable ground state for this striped structure, we start with a structural optimization of the slab to relax all forces and thus locate a total energy minimum. The resulting atomic structure is displayed in Figure 5. In Figure 5a we show a side view of the *ab initio* relaxed structure. Here, the Bi (Se) atoms are represented by purple (green) balls, clearly illustrating the seven primitive unit cell wide surface QL on top of the bulk eight unit cell wide QLs. We find that the obtained relaxed lattice structure is corrugated with moderately large distortions in the *c* direction. To quantify these distortions, we introduce the parameter δ measuring the out-of-plane (*c* direction) distortion of each Se atom in the outermost layer, as schematically illustrated in Figure 5b. We obtain these distortions relative to the Se atom having the least out-of-plane distortion within each layer and plot the result in Figure 5b as a function of distance along the *a* direction, along with a spline fit (solid line). We find that the maximum distortion, δ_max_, occurs at about half the supercell lattice distance from the minimum distortion, δ_min_, with a total distortion of δ_max_ – δ_min_ = 0.027 nm. Considering δ_max_ and δ_min_ as the top and valley positions, respectively, sketched in Figure 1c, the simulation gives 3.4 nm as the distance between two adjacent stripes, a result well within the experimental uncertainty of the measurements reported in Figure S2b for Bi_2_Se_3_ deposited on a Si substrate.

Finally, we discuss the electronic properties of the obtained 1D moiré striped lattice structure. The main motivation is to shed light on the conductance measurements reported in Figure 1d. For this purpose, we first calculate the LDOS corresponding to each atom (both Bi and Se) in the surface QL. We then focus on the regions around δ_max_ and δ_min_ in the surface QL by selectively summing the LDOS of the atoms up to a depth (in the *c* direction) of three atoms, such that we include a single atom within each *a*–*b* plane layer in each max/min region. Figure 5c shows this summed LDOS with blue and red lines displaying the LDOS in the δ_max_ and δ_min_ regions, respectively. As a comparison, we also plot the similarly summed LDOS of the surface QL of pristine Bi_2_Se_3_ (green line). Here we have located the bulk energy gap (pink background) of both the pristine and striped Bi_2_Se_3_ slabs and used that to align the LDOS spectra. Having aligned the bulk energy gaps, we see that also the Dirac points of both the pristine and striped slabs are located at zero energy. The surface state spectra in Figure 5c display several interesting features. The pristine Bi_2_Se_3_ displays a linear LDOS profile throughout the bulk energy gap, due to the Dirac surface state. In the striped slab we find that the Dirac point is still at zero energy throughout the surface, but the linear LDOS regime is now much narrower, only existing in a small low-energy window around the Dirac point (between −0.02 and 0.05 eV). The slope of the LDOS in this linear regime is also larger, indicating that the stripe distortion has renormalized the velocity in the Dirac surface state to significantly smaller values, and with a large spatial variation between the δ_max_ and δ_min_ regions. In particular, the Fermi velocities, as calculated by the slopes of the LDOS^26^ in top and valley positions, are reduced by 48% and 32%, respectively, with respect to the value calculated for the pristine structure. Considering that the stripes are likely present at all surfaces of nanowires and films, this result directly gives a tentative explanation for the experimentally measured reduced Fermi velocity in Bi_2_Se_3_ with respect to the expected velocity.^16^ Beyond the linear LDOS regime, we find a rather irregularly varying LDOS within the bulk energy gap, in both the δ_max_ and δ_min_ regions. Notably, the LDOS is about 3 times larger in the δ_min_ region compared to the δ_max_ region and notably higher than that on the pristine surface. This large variation in the LDOS along the *a* direction can account for the differences in the electrical conductance observed in experiments going through top to valley, as seen in Figure 1d.

The features observed in Figure 5c for the striped Bi_2_Se_3_ surface have many similarities with theoretically calculated spectra of TI surface states in artificially created 2D moiré superlattices,^8,9^ while the stripe formation in our experiment and calculations is clearly a single-directional modulation. For example, the Dirac point has been shown to be preserved even in a 2D moiré superlattice, but with the velocity renormalized to smaller values and the linear DOS energy regime significantly narrower, features we also clearly observe in Figure 5c. Moreover, a 2D moiré superlattice has been shown to create multiple additional Dirac points and van Hove singularities at higher energies, but still within the bulk energy gap, generating many peaks and dips in the DOS. Again, this is reminiscent of the DOS seen in the surface layer of the striped Bi_2_Se_3_ in Figure 5c, especially considering the finite energy resolution of the large-scale *ab initio* calculations. We here point out that we also find a clear spatial dependence in the LDOS, including different Dirac velocities in the top and valley positions.

The experimental results and DFT calculations demonstrate the intrinsic nature of the 1D moiré stripes as being due to a unidirectional strain mechanism of the Bi_2_Se_3_ top layer. In fact, strain in the top layer as large as 17%^27^ and differential conductance modulation^28^ in TIs are well-known phenomena related to two- or three-directional effects. They are usually ascribed to particle contamination (mainly carbon and oxygen) or to the substrate mismatch. However, being randomly distributed on the sample surface, contaminants and/or vacancies cannot be responsible for the uniaxial strain. On the other hand, this can be conjectured on the basis of the growth mechanism for both nanobelts and thin films. In the case of the nanobelts, the deposition process happens at high temperatures and under the streaming of the atomic species, which nucleates into a stable bulk Bi_2_Se_3_ lattice structure (see Methods, the Supporting Information, and refs (14 and 16) for details). The growth process suddenly stops by natural cooling, and a abrupt interruption of the flux streaming occurs with a consequent reduction of the deposited species on the belt surface. This lack of material and temperature reduction freezes the coalescent layer as shown in Figure S5b. Consequently, a reconstruction of the lattice structure in the direction perpendicular to the coalescent front layer is expected, with the formation of the strained lattice parameter along this direction and resulting stripe formation. A similar mechanism can be reasonably expected also in the case of thin films because the deposition method follows the same principles of the nanobelt growth being achieved by the streaming of the species on the substrate.^14^ The coalescent front layers are, in this case, well evidenced by STM measurements reported in Figure S5c, showing large moiré striped terraces frozen at the end of the growth process. It is reasonable, at this point, to argue that different matching between the two topmost layers could possibly happen during the growth dynamic with the consequent formation of stripes with different periodicities. In the case of the films, the granularity of the substrate surface also plays an important role in confining the coalescent front layer, with the result of breaking any possible long-range parallel and periodic modulation in the stripes, as better evidenced for the Pt substrate in Figure S4a. Under this aspect, this can be considered as the cause of the average lower periodicity of the moiré stripes in films deposited on Pt substrates. All of these considerations suggest that proper control of the growth parameters such as temperature, Ar pressure, and flow rate could help in influencing the growth mechanism. It is plausible to assume that, by filling the chamber with a different Ar pressure and streaming and by ramping down the temperature at a programmed rate, one can control the amount of material available for the growth of the last layer and therefore the effective strain. In the case of thin films, the same mechanism can be triggered by regulating the pumping speed during the deposition. Moreover, the use of vicinal substrates can influence the direction of the coalescent front layer, giving rise to a preferential orientation of the striped structure.

## Conclusion

In conclusion, STM and HRTEM measurements have shown well-defined and extended 1D moiré stripes on Bi_2_Se_3_ thin films and nanobelts, which cannot be ascribed to a mismatch with the substrate lattice. Our STS data show an enhanced LDOS that is modulated across the moiré stripe profile. These structural and electronic observations prompt a need to find growth conditions to engineer “*in situ*” strain-induced moiré stripes extending over an even larger area of the sample surface. This would give access to interacting instabilities and a not yet investigated phenomenology related to the topological protected Dirac cones, in addition to the emergence of low-energy van Hove singularities. In particular, the greatly enhanced surface Dirac state density can lead to a topological superconducting transition in an exponential fashion, making surface superconductivity in Bi_2_Se_3_ more viable.

## Methods

### Fabrication

The fabrication process is detailed in ref (14) The process consists of two steps: one producing nanobelts and the following one producing the films. During the first step, Bi_2_Se_3_ powder is evaporated at 585 °C in a quartz tube at a pressure of 5 mbar and condenses on the substrates in the form of nanobelts when the temperature is reduced from 540 to 500 °C under 50 sccm Ar streaming at a pressure of 35 mbar. The process stops when the temperature is below 500 °C. During the second step, Bi_2_Se_3_ films are deposited on substrates at 10^–3^ mbar and at a temperature below 100 °C by using the Bi_2_Se_3_ species condensed on the quartz tube during the first step eventually enriched with Se added in the quartz tube to compensate for its volatility.

Both Si (lattice parameters *a* = *b* = *c* = 0.543 nm) and Pt (lattice parameters *a* = *b* = *c* = 0.392 nm) substrates were obtained from the same 7 × 5 mm^2^ commercial Si(001) single crystals, the latter one by thermally depositing 150 nm of Pt upon a partial area 3 × 5 mm^2^ of the Si surface previously buffered with 300 nm of SiO_2_.^14^ The substrates were chemically etched for 30 s in standard 5% HF solution to remove native oxide from their surfaces before the deposition. No reconstruction of the Si surface under a vacuum or ion milling process was performed for further cleaning of the substrate surface before the film deposition. XRD shows the preferred (111) orientation for this Pt layer, which we labeled as the Pt(111) substrate throughout the text. All of the Bi_2_Se_3_ samples were deposited during the same deposition run with the substrates positioned at different distances from the source materials in order to achieve the desired final thickness, measured by X-ray reflectivity on previously deposited test samples.^14^ Due to the low-vacuum deposition system, a native oxide layer is possibly formed on the Si and Pt surfaces despite the HF etching performed right before the deposition.

A Philips XPERT MRD high-resolution diffractometer equipped with a 4Ge monochromator and a 3DPixcell detector was used for thickness measurements and for studying the crystal structure of the obtained samples.

### STM and STS Measurements

For the STM measurements on thin films, a combined STM/AFM Omicron instrument operating under ultrahigh vacuum at room temperature was used. The bias voltage was in the range −0.5 V and 0.6 V, and the typical current was *I* = 0.5 nA. STS was performed by repeatedly mapping the same STM-investigated areas acquiring current voltage (*I–V*) characteristics using different STM tip–sample gap voltages in the range −0.74 V and 0.45 V. No remarkable dependence on the experimental parameters was observed in both STM and STS analyses. Bi_2_Se_3_ deposited on glass was not analyzed by STM because of the insulating character of the substrate.

For STM measurements on nanobelts, they were mechanically removed from their growth substrate, transferred onto an *n*-doped Si substrate, and loaded into the UHV chamber of an Omicron VT STM instrument operated at room temperature. Several cycles of 10 min Ar^+^ ion sputtering and 10 min annealing were performed in order to remove the surface oxide that had formed during sample transport through air, using sputtering parameters of up to 1 kV acceleration voltage and up to 20 mA emission current at an Ar pressure of 1 × 10^–6^ mbar and annealing temperatures of up to 400 °C. Figure 2a shows an STM overview image of a part of a deposited nanobelt prior to oxide removal. After sputtering and annealing, an atomic resolution could be obtained in STM images from sufficiently flat and clean areas of the surface, as demonstrated in the inset of Figure 2a, with a lateral distance between the atoms of 0.4 nm.

### TEM Analysis

The morphology of Bi_2_Se_3_ nanobelts was first analyzed by a ZEISS Supra 25 SEM instrument, operating at low energy (1 keV) to enhance surface features. Then, the nanobelts were scratched onto a 3 mm Au grid with a holey-carbon layer to be analyzed by a JEOL 2010F TEM instrument, operating at 200 keV. The latter was used also for structural investigations via electron diffraction (not shown) by using the smallest selected area aperture (10 μm in diameter). Further information was gathered by analyzing the fast Fourier ransform of high-resolution electron micrographs (HREMs). Both diffraction patterns and HREMs have been analyzed by using Gatan Microscopy Suite software.

### DFT Theoretical Calculations

Electronic structure calculations were performed on a slab six Bi_2_Se_3_ quintuple layers (QL) thin, using density functional theory (DFT), as implemented in the Vienna *ab initio* simulation package (VASP). Each QL of Bi_2_Se_3_ consists of five alternating layers of bismuth and selenium atomic layers, while the QLs are separated from each other by a van der Waals gap. We repeat the primitive Bi_2_Se_3_ unit cell along the in-surface *a* direction for eight periods, except for the surface QL on each side of the slab. The surface QL is instead uniformly stretched in the *a* direction, such that it contains only seven periods of the primitive Bi_2_Se_3_ unit cell, creating an overall stripe structure consistent with the experimental measurements in Figures 1 and 2 This results in a surface QL being rather substantially stretched, or distorted, compared to the bulk QLs.

We used the DFT-D3 method with Becke–Johnson damping in order to account for the van der Waals interactions between the QLs, using a kinetic energy cutoff of 300 eV. We further used *k*-point meshes (*k*_*a*_ × *k*_*b*_ × *k*_*c*_) of 1 × 8 × 1 and 4 × 32 × 1 for the structural and electronic optimizations, respectively. Both meshes were checked in terms of total energy convergence. For the structural optimization we performed the relaxation until an energy convergence threshold of 10^–5^ eV was obtained and the forces on each atom were less than 30 meV/Å. For the electronic optimization, we used an energy convergence threshold of 10^–7^ eV for obtaining the ground state.

## Supporting Information Available

X-ray reflectivity for thickness measurements and X-ray diffraction for structure analysis, stripe modulation statistics on Bi_2_Se_3_ deposited on Si and Pt substrates, direct current–voltage characteristics on different top and valley positions of the stripes, STM and STS measurements on Bi_2_Se_3_ deposited on Pt substrates, SEM images of Bi_2_Se_3_ nanobelts, and moiré patterns generated by bidimensional strain and by twisted layers (PDF)
