# Supplementary material for: Nanometric Moiré Stripes on the Surface of Bi2Se3 Topological Insulator
Source: ACS Nano. 2022 Sep 13;16(9):13860–8. doi: 10.1021/acsnano.2c02515 (PMC9527797; doi:10.1021/acsnano.2c02515)
Supplement: Supplementary file 1 — nn2c02515_si_001.pdf [file nn2c02515_si_001.pdf]

Supporting information

Nanometric moiré stripes on the surface of Bi_2Se_3 topological insulator

Matteo Salvato*,[§] Maurizio De Crescenzi,[§] Mattia Scagliotti,[§] Paola Castrucci,[§] Simona Boninelli,[illegible] Giuseppe Mario Caruso,[illegible] Yi Liu,[¥] Anders Mikkelsen,[¥] Rainer Timm,[¥] Suhas Nahas,[ǂ] Annica Black-Schaffer,[ǂ] Gunta Kunakova,[ǂ,˜] Jana Andzane,[ǂ] Donats Erts,[ǂ] Thilo Bauch,[˜] and Floriana Lombardi*.[˜]

[§]*Dipartimento di Fisica and INFN, Università di Roma "Tor Vergata", 00133 Roma, Italy*

[illegible]*CNR-IMM, Strada VIII 5, 95121 Catania, Italy*

[¥]*Division of Synchrotron Radiation Research, Department of Physics and NanoLund, Lund University, 221 00 Lund, Sweden*

[ǂ]*Department of Physics and Astronomy, Uppsala University, Box 516, 75120 Uppsala, Sweden*

[ǂ]*Institute of Chemical Physics, University of Latvia, LV-1586 Riga, Latvia*

[˜]*Quantum Device Physics Laboratory, Department of Microtechnology and Nanoscience, Chalmers University of Technology, 41296 Goteborg, Sweden*

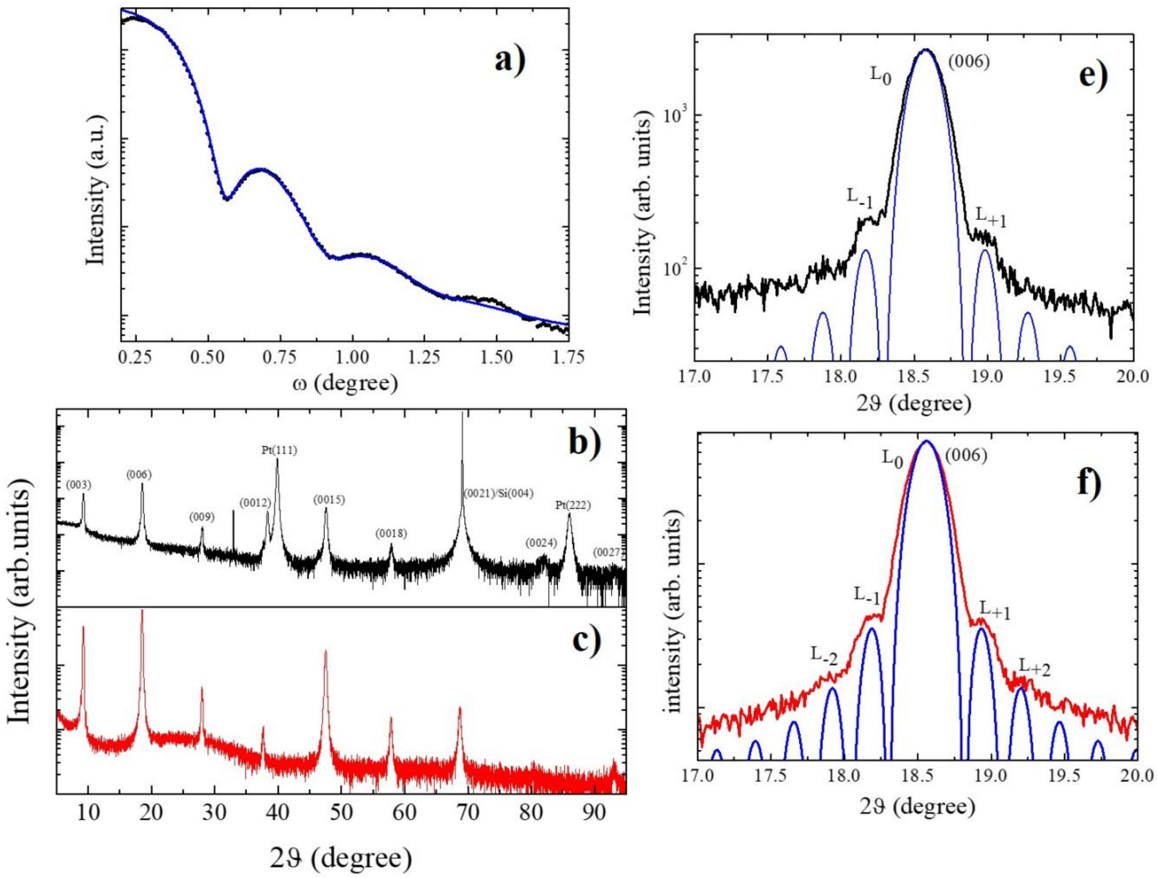

Figure S1. a) Typical x-ray reflectivity of Bi_2Se_3 thin films. The data points refer to the experimental data and the blue line is a fit to the data giving 10.5 nm as thickness of the film. b) XRD spectrum of Bi_2Se_3 film 20 nm thick deposited on Si and Pt substrates. The label refers to the Bi_2Se_3 reflections according to the rhombohedral R-3m indexing. c) same as b) but deposited on amorphous glass. e) and f) are (006) reflections of Bi_2Se_3 films deposited on Si and on glass substrates respectively showing Laue reflections. The blue lines are qualitative simulation obtained by considering 9- and 12-unit cells in the Bi_2Se_3 structure, respectively.

Figure S1. (a) shows x-ray reflectivity of a sample deposited on a Si substrate. The red line is the best fit to the experimental data obtained by using the Nelder-Mead algorithm in REFLEX package. The film thickness for this sample, evaluated by the fitting procedure, is 10.3 nm obtained considering a substrate roughness of 0.14 nm. The chi-square of the fit was 0.01.

The crystal structure and the orientation of the obtained samples were studied by x-ray diffraction (XRD). The XRD data are shown in Figure S1 b) where, apart from the Pt(111) and the Si(004) reflections, only the Bi_2Se_3 (0,0,3*l*) peaks appear, confirming that the films grow with the *c* axis oriented perpendicular to the substrate plane. The value estimated for the ***c*** axis is *c*=2.86 nm in very good agreement with the literature. The same ***c***-axis length and the same orientation was obtained for the samples deposited on glass substrates (Figure S1-c) which have an amorphous structure as confirmed by the absence of any reflection different from that ascribed to the Bi_2Se_3. A limited range of the same spectra related to the (006) reflections is reported in Figure S1e and S1f, respectively. In both the cases, the main (006) reflection is surrounded by the satellite Laue reflections which give a

strong indication that the films are formed by an integer number of unit cells. This is confirmed by the simple qualitative simulation reported in red in the same figure and obtained by the modulation function $sin^2(0.5Nqc)/sin^2(0.5qc)$ assuming N=9 and N=12 unit cells for Bi_2Se_3 deposited on Si and on glass substrate respectively (in the expression q is the x-ray wavevector). The presence of the Laue reflections and the same c-axis orientation is a strong indication that the Bi_2Se_3 lattice structure and orientation are not affected by the substrate, as expected for van der Waals structures, and that the growth proceeds following the stacking of QL blocks along the direction perpendicular to the substrate surface.

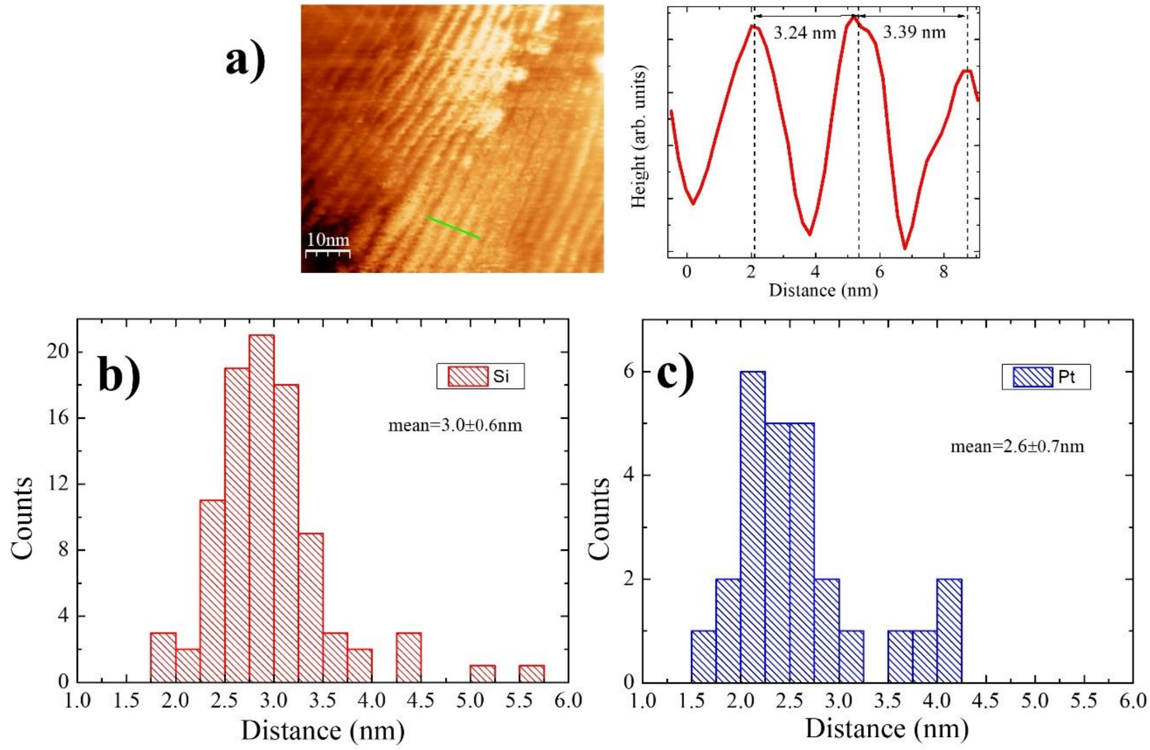

Figure S2 a) stripes observed on Bi_2Se_3 film deposited on Si substrate and line profile measured along the transverse green line showing an average distance of 3.3 nm; b) histogram consisting of 97 stripe distance measurements on different patterns acquired on Bi_2Se_3 thin films deposited on Si substrates; c) same as b) consisting of 29 experimental stripe distances measured for films deposited on Pt substrates. The calculated average distance is 3.0±0.6 nm and 2.6±0.7 nm for Bi_2Se_3 deposited on Si and Pt respectively where the uncertainty is the standard deviation of the experimental data

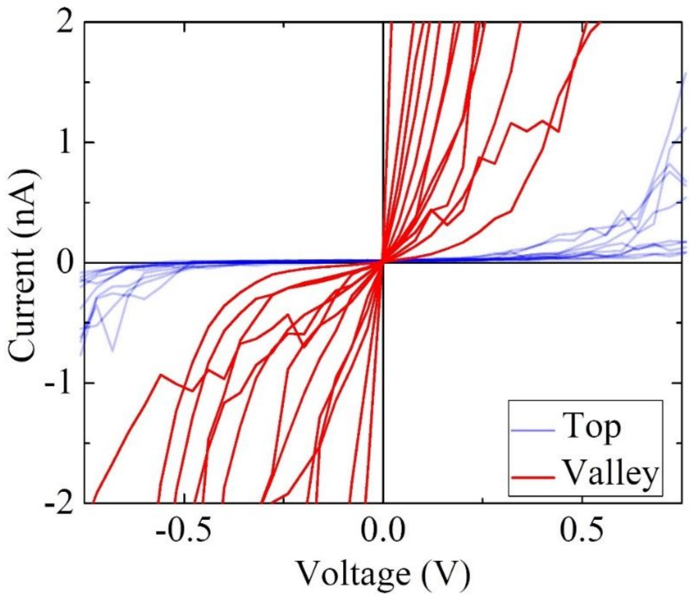

Figure S3. Direct current-voltage (*I-V*) measurements performed in different areas of the 10 nm thick Bi_2Se_3 film. Blue lines are acquired on the top of a stripe while red lines are measured between two adjacent stripes (valley). The sizable different slopes between the two characteristic groups give rise to the marked difference in the conductance reported in Fig.1d of the main text.

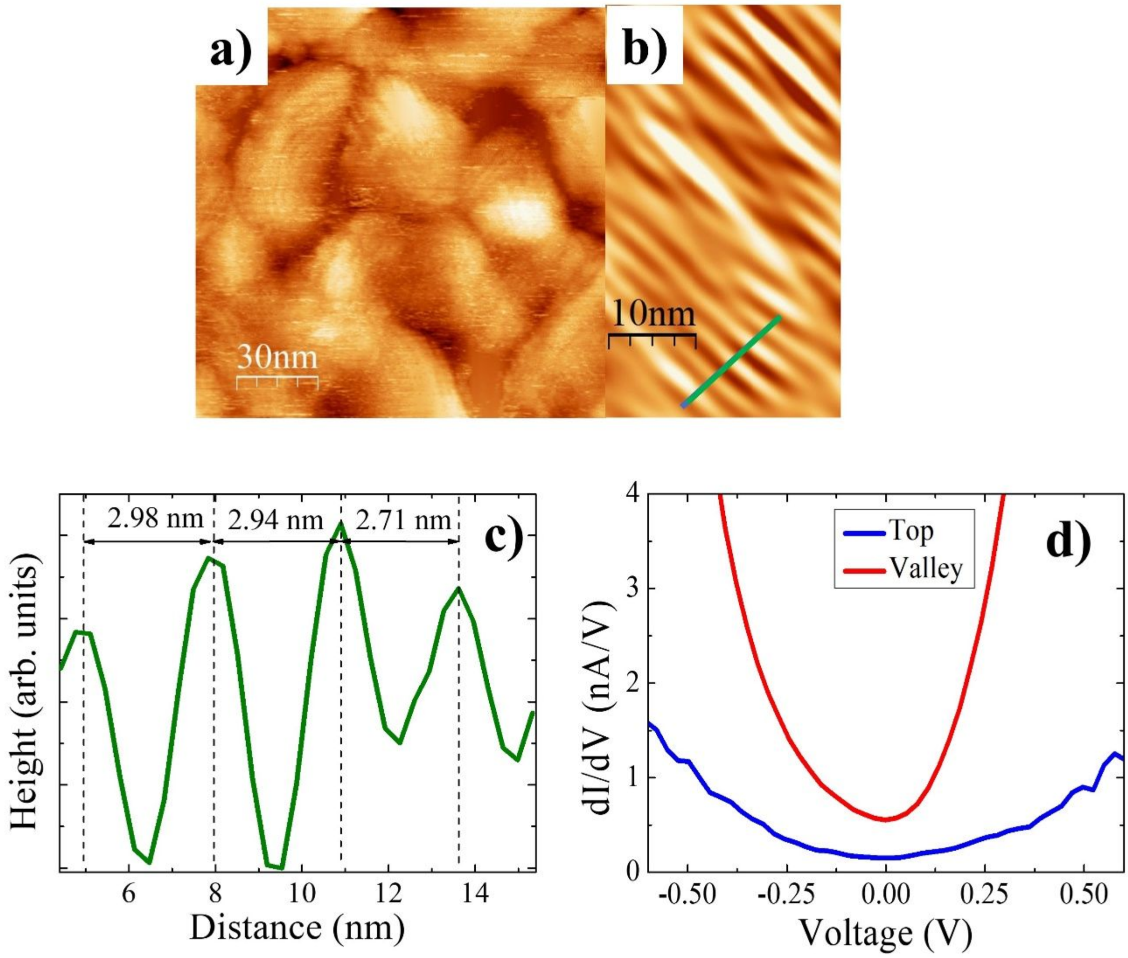

Figure S4. a)-b) STM images at different resolutions of a 10 nm thick Bi_2Se_3 film deposited on a Pt substrate. The green line profile in b) is reported in c) where an average distance between two adjacent stripes of 2.9 nm is measured. d) STS measurement performed on the top (blue) and valley (red) of two adjacent stripes. V=0 corresponds to the Fermi level. Experimental parameters for STM are V=0.5 V, I=0.5 nA; for STS V bias step=40 mV.

Figure S4a shows the STM image of a 10 nm thick Bi_2Se_3 film deposited on Pt(111) substrate. The surface morphology shows the presence of grains with moiré stripes confined inside their perimeter. The presence of grains instead of large terraces, as observed for Si, can be ascribed to the different substrate morphology, being Si(001) a single crystal while Pt has been obtained by thermal evaporation on the SiO_2 buffer layer. Figure S4b shows the STM analysis on one such grain and Figure S4c shows the line profile measured along the green line. As in the case of Si substrates, the distance between the stripes is not constant. The different distances measured on several samples have been collected for the statistics shown in Figure S2c. Figure S4d shows the differential conductance vs. bias voltage on the top and between two adjacent stripes (Valley). Also in this case, a higher metallicity is measured between the stripes as evidenced in the figure by the different values measured for the *dI/dV* around the Fermi level.

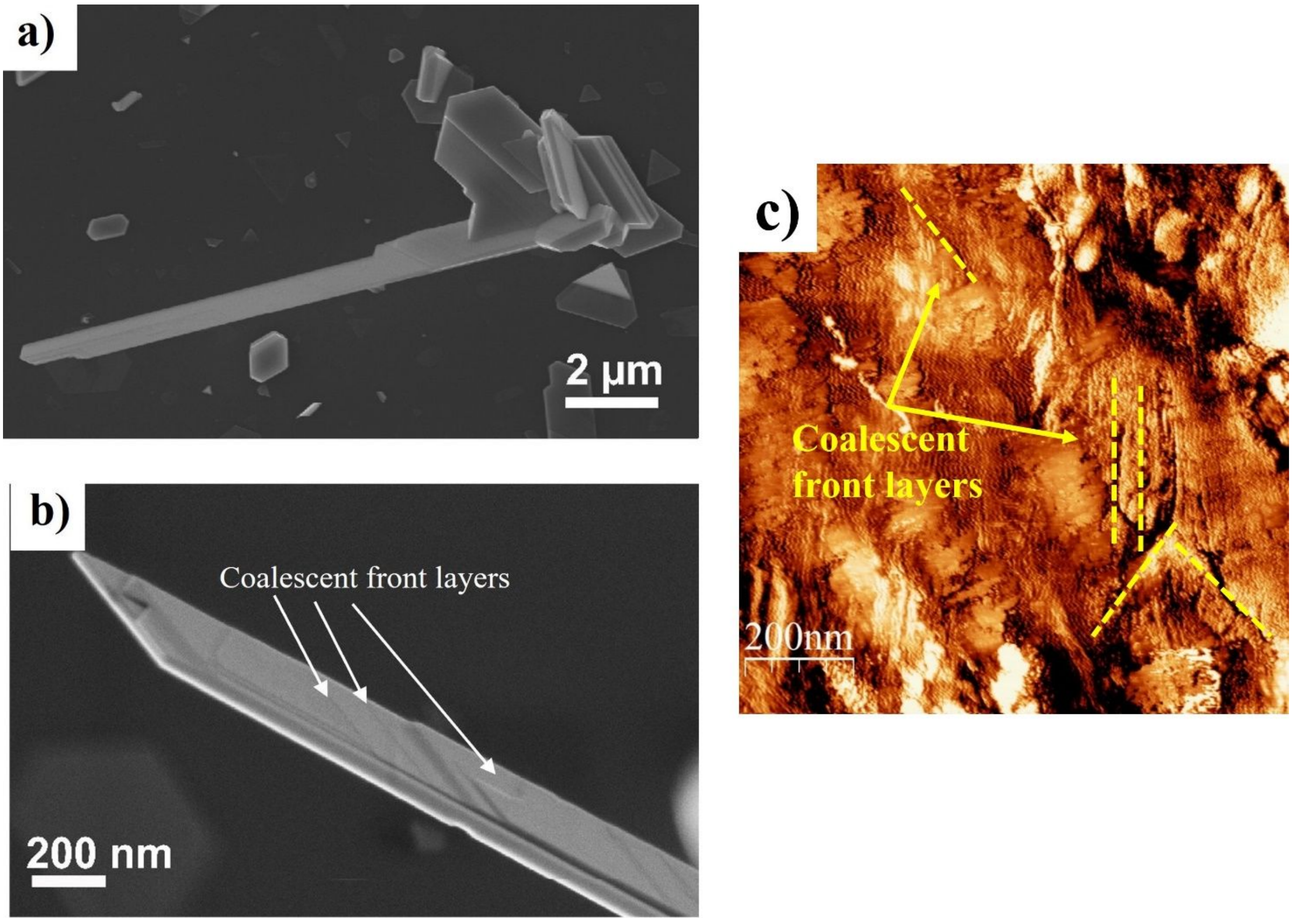

Figure S5. a) SEM micrograph of a Bi_2Se_3 nanobelt growing from a multifaced platelet. b) Detailed view of the step-like structure of the nanobelt tip showing coalescent front layers forming at the end of the growth. c) 1µm x 1µm STM image of a 10 nm thick film deposited on Si (001) showing large striped terraces delimited by coalescent front layers (some evidenced by dashed yellow lines).

The mechanism of the growth of the nanobelts is better understood by referring to F**igure S5**a and S5b. Figure S5a shows a scanning electron microscope (SEM) image of a nanobelt attached to a Bi_2Se_3 nanoplatelet[14]. This is formed during the first instants of the growth and works as a seed for the nanobelt which grows thanks to the Bi_2Se_3 species transported by an Ar streaming at low pressure. During this phase, the nanobelt grows in a layer-by-layer fashion where the front layers spread in two dimensions but mostly along the longitudinal direction. The sudden reduction of the temperature and the Ar streaming interrupts the growth process freezing the front layers as shown in Figure S5b. Consequently, a strain in the top layer is generated giving rise to a lattice mismatch with the underlayer which is evidenced by the moiré stripe formation. In a similar way, the films are deposited on a substrate in a second step[14] when the high temperature Bi_2Se_3 species are transported under vacuum on substrates maintained at room temperature. As in the case of nanobelts, the vaporized Bi_2Se_3 species freeze on the substrates forming coalescent front layers with strained lattice parameter and consequent stripe formation.

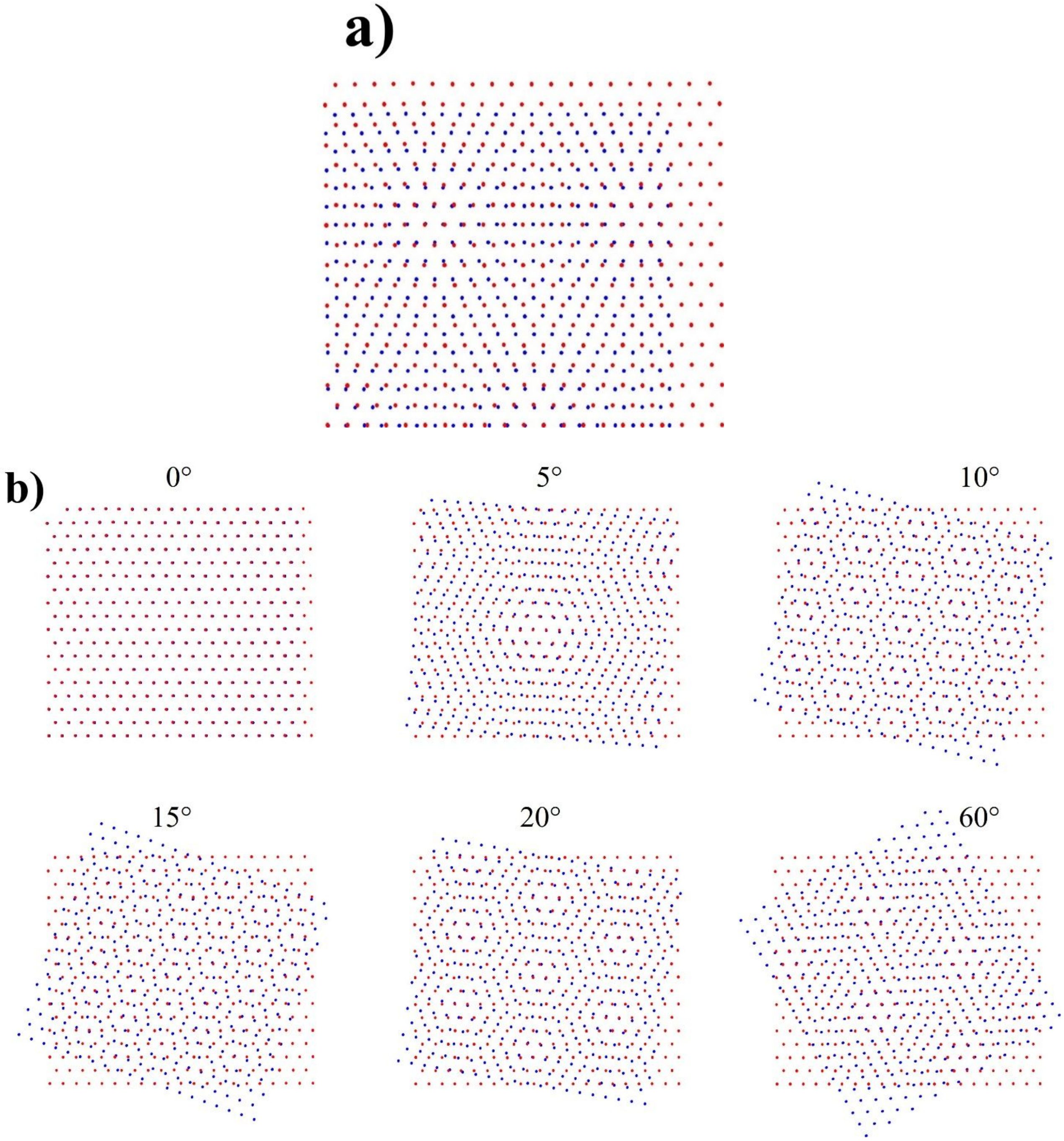

Figure S6. Some representative moiré patterns obtained by a) a bi-directional strain and b) by the twists between two overlapped triangular layers at indicated angles.

In **Figure S6** red and blue spots represent two hexagonal overlapped layers. In Figure S6a one of the layers (the red one) is strained along two directions. Figure S6b shows the same layer twisted by different angles. In any case, no stripes appear as a final pattern. The only possibility to observe parallel stripes is the uniaxial strain as reported in Figure 4a of the main text.
